# The Interplay Between Vitamin D Deficiency, Iron Status, and Anemia Risk in Moroccan Women of Reproductive Age: A Cross-Sectional Analysis

**DOI:** 10.3390/epidemiologia5040055

**Published:** 2024-12-19

**Authors:** Noura Zouine, Ilham Lhilali, Lode Godderis, Adil El Midaoui, Samir El Jaafari, Younes Filali-Zegzouti

**Affiliations:** 1 Cluster of Competency “ Environment and Health”, Faculty of Sciences, Moulay Ismail University, Meknes 50000, Morocco; i.lhilali@edu.umi.ac.ma (I.L.); s.eljaafari@umi.ac.ma (S.E.J.); y.filalizegzouti@fste.umi.ac.ma (Y.F.-Z.); 2 Higher Institute of Nursing and Health Professions of Fes-Meknes Annex, Meknes 50000, Morocco; 3 Health and Environment Unit, Faculty of Medicine, KU Leuven, 3000 Leuven, Belgium; lode.godderis@kuleuven.be; 4 IDEWE, External Service for Prevention and Protection at Work, 3001 Heverlee, Belgium; 5 Faculty of Sciences and Techniques, Errachidia, Moulay Ismail University of Meknes, Meknes 52000, Morocco; adil.el.midaoui@umontreal.ca; 6 Department of Pharmacology and Physiology, Faculty of Medicine, University of Montreal, Montreal, QC H3C 3J7, Canada; 7 BASE Laboratory, FSM-FSTE, Moulay Ismail University of Meknes, Meknes 50000, Morocco

**Keywords:** vitamin D, vitamin D deficiency, iron deficiency, iron deficiency anemia, anemia, erythropoiesis, dietary intake, women of reproductive age

## Abstract

**Background:** Vitamin D and iron deficiencies are prevalent among Moroccan women of reproductive age (WRA). Research suggests that Vitamin D deficiency (VDD) may impair iron bioavailability, potentially leading to iron deficiency (ID) and anemia. Objectives: This study investigates associations between vitamin D status, iron levels, and anemia risk in WRA, aged 18–49, from Meknes, Morocco. **Methods:** A cross-sectional study was conducted among 463 participants, measuring serum 25(OH)D, blood count parameters, iron, ferritin, C-reactive protein, and creatinine. Lifestyle factors, including dietary intake, sun exposure, and physical activity, were assessed through validated questionnaires, and anthropometric data were collected. Linear and logistic regression models analyzed associations, while ROC analysis evaluated VDD’s predictive accuracy for ID and anemia. **Results:** VDD (25(OH)D < 20 ng/mL) was significantly associated with reduced hemoglobin, hematocrit, red blood cells, and ferritin (all *p* < 0.01), indicating vitamin D’s role in erythropoiesis and iron storage. Multivariate logistic regression showed that VDD increased the risk of anemia (OR: 7.17, 95% CI: 3.19–19.28, *p* < 0.001), ID (OR: 2.20, 95% CI: 1.32–3.77, *p* = 0.007), and IDA (OR: 4.10, 95% CI: 1.73–12.08, *p* = 0.004). Dietary iron intake was inadequate, showing minimal protective effects against anemia and ID (β(SE): −0.08(0.03), *p* = 0.030 and β(SE): −0.05(0.02), *p* = 0.037). **Conclusions**: VDD is a significant risk factor for impaired iron status and anemia in Moroccan WRA, highlighting the need for targeted nutritional interventions and further research.

## 1. Introduction

Vitamin D deficiency (VDD) and anemia continue to affect millions of women worldwide, predominantly in low-and middle-income countries [1,2], resulting in impaired economic productivity [1,3], increased morbidity and all-cause mortality [4,5], and risk of adverse outcomes for mothers and newborns [6,7].

Anemia occurs when red blood cells and hemoglobin levels are too low to meet the body’s oxygen needs [8,9]. Its etiology is multifactorial, involving nutritional deficiencies (e.g., iron, vitamins A and B12, folate, and riboflavin), genetic conditions (e.g., sickle cell disease and thalassemia), and inflammation from infectious or chronic diseases [10]. Iron deficiency (ID) is the primary common cause, accounting for 50% of all anemia cases among women in developed countries [9]. Women of reproductive age (WRA) are at particularly increased risk of ID due to physiological changes and increased nutrient requirements during pregnancy or growth periods [9]. Despite awareness of causes and treatments, anemia prevalence in WRA has remained nearly unchanged since 2000, affecting almost one-third of non-pregnant WRA (ages 15–49) worldwide [9]. The WHO established a Global Nutrition Target to reduce anemia in WRA by 50% by 2025, which was later extended to 2030 [9].

Vitamin D is a secosteroid with two primary forms: vitamin D2 (ergocalciferol) and vitamin D3 (cholecalciferol) [11]. Approximately 80% of vitamin D3 is synthesized in the skin through UVB exposure (295–315 nm), with the remaining 20% obtained from dietary sources [12]. Vitamin D2 is produced in plants, fungi, and yeast when exposed to UV, while animal sources like fatty fish, cod liver oil, and egg yolks provide vitamin D3 [13]. Supplements and fortified foods also contribute to vitamin D intake [12,13]. All forms are converted first to 25(OH)D in the liver by 25-hydroxylase enzymes and then to the active form, 1,25-dihydroxyvitamin D (calcitriol), in the kidneys by 1-alpha-hydroxylase (CYP27B1) [14].

Vitamin D functions in the body as a fat-soluble vitamin and prohormone with effects on multiple systems, including bone health, calcium and phosphate metabolism, and gene expression [14,15]. Vitamin D actions through a single vitamin D receptor (VDR), which also has been identified in the ovaries and the endometrium, among other human female reproductive tissues [16,17].

Vitamin D has drawn attention to its potential role in anemia and iron regulation. Studies suggest that vitamin D may reduce anemia by directly stimulating erythroid precursors [18,19], as the vitamin D receptor (VDR) is present in bone marrow at concentrations 100 times higher than in plasma [20]. Bacchetta et al. identified a VDR binding site on the hepcidin promoter, indicating that vitamin D may downregulate hepcidin, the key hormone controlling systemic iron homeostasis [21]. Hepcidin degrades ferroportin, thus limiting iron absorption and release from storage [21]. Additionally, vitamin D’s anti-inflammatory effects may lower hepcidin by reducing pro-inflammatory cytokines (e.g., IL-6, IL-1β), which otherwise restrict iron availability and may lower hemoglobin [22], which otherwise elevate hepcidin levels, restricting iron availability and potentially lowering hemoglobin concentrations [23]. This anti-inflammatory role of vitamin D is particularly relevant to anemia of inflammation [24].

The association between low 25(OH)D levels and disruptions in both iron status and hemoglobin concentrations has been demonstrated across various populations [5,25,26,27,28,29,30,31,32]. Trials results showed that vitamin D intake increases hemoglobin, erythrocyte, and iron levels [33], and improvement in vitamin D status to be >20 ng/mL is positively correlated to transferrin saturation (a marker of iron supply to tissues) in women [28]. An increase in vitamin D concentration after intramuscular iron treatment in infants was also observed in other investigations [34]. This suggested a reciprocal interplay between vitamin D and iron [31,32] that may be beneficial in both VDD and iron deficiency recovery.

VDD affects over one billion people globally, regardless of age, sex, ethnicity, or geographic location [35,36]. While no consensus exists, VDD is defined by a serum level of 25-hydroxyvitamin D (25(OH)D) < 20 ng/mL (50 nmol/L) [36].

Despite an average of 8 h of daily sunshine, reaching up to 9.5 h in desert areas [37], Morocco shows significantly lower vitamin D levels than other regions in Africa [38], highlighting unique local challenges.

Low vitamin D levels are reported across various groups in Morocco, including pregnant women, newborns [39], rural school-aged children [40], adults [41,42], and older people [43]. In postmenopausal women, deficiency is associated with conditions affecting bone and mineral density, such as systemic scleroderma and osteoporotic fractures [44,45], and in men with ankylosing spondylitis [46].

Studies in Morocco’s sunniest regions report high hypovitaminosis rates (25(OH)D < 30 ng/mL) of approximately 90–100%, particularly among women [47,48,49,50]. The 2019 National Nutrition Survey found that 78% of WRA have 25(OH)D levels below 20 ng/mL, with one-third severely deficient [51]. Contributing factors were studies in disparate studies and included reduced daily outdoor sunlight exposure [42,48,51,52], low sun exposure scores from indoor habits and sun protection use (e.g., sunscreen) [49,50], restrictive clothing [42,48], high rate of obesity and adiposity [49,50,53,54], low calcium [42] and vitamin D intake [49], urbanization [38,49,51,55], and younger age [50,51]. Additionally, higher melanin levels in darker skin across large regions of the country may reduce cutaneous UV absorption [56], potentially lowering vitamin D synthesis [38,48].

To address nutrient deficiencies, Morocco has prioritized fortifying foods with vitamin D and iron [57]. Vitamin D supplementation programs target children at birth and 6 months to reduce nutritional rickets, while pregnant women receive iron and vitamin D2 to combat maternal anemia and improve birth outcomes [58]. Nevertheless, anemia rates in WRA rose from 32.9% in 2006 to 34.4% in 2019, with 49.7% of cases due to iron deficiency anemia (IDA) and one-third of non-pregnant WRA have remained affected by iron deficiency since 2000 [51]. High rates of overweight and obesity alongside these micronutrient deficiencies indicate a national nutritional transition, creating a double burden of malnutrition [51]. These dynamics suggest that vitamin D and iron deficiencies in WRA may continue to present persistent public health concerns.

This is the first study in Morocco to examine associations between VDD, iron status, and anemia in WRA. Using an integrated assessment approach that considers known determinants, we investigated whether VDD is a modifiable risk factor contributing to iron deficiency and anemia rates. Findings could support targeted prevention strategies and inform trials on vitamin D supplementation as a preventive or therapeutic intervention in this vulnerable population.

## 2. Materials and Methods

### 2.1. Study Design and Population

This was a cross-sectional study involving WRA from the Meknes prefecture (Morocco) who presented for vitamin D blood analysis prescribed by a clinician in a private medical clinic and certified laboratory that participated in the study. Enrolment was conducted between February and December 2022.

**Figure 1:**
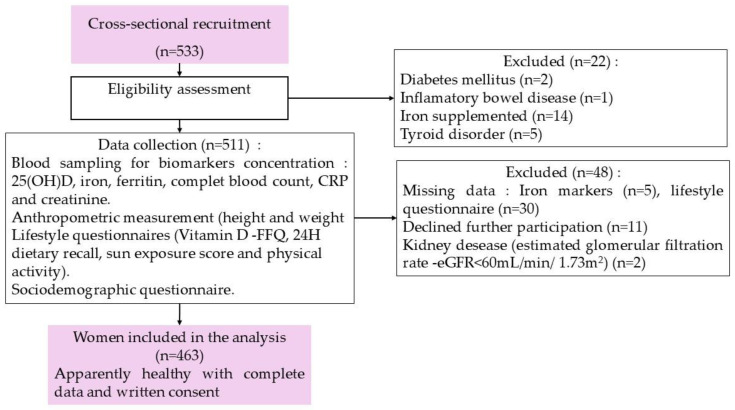
Participant flow chart.

The sample size was obtained by Cochran’s formula n = Z^2^ pq/e^2^ [59], where Z is the standard normal variate at a confidence interval of 95% = 1.96 and *p* is the prevalence of vitamin D deficiency (25(OH)D < 20 ng/mL) = 78.8%, which was stated in the National Nutrition Survey (ENN 2019) [51], q is 1 − *p*, and e is the margin of error = 0.05. Hence, a minimum number of 256 participants was required to obtain statistically representative data. To increase statistical power and consider the completion of response, a total of 533 women participants were recruited for the study, of which 463 consented to conclude assessments with the research team and were eligible for our inclusion criteria (Figure 1).

Women were eligible if they were aged 18 to 49 years, apparently healthy, and were able to provide consent to participate in the study. Women were excluded if they reported any significant medical conditions that affect hemoglobin, iron, or 25(OH)D serum concentrations, such as abnormal renal/liver function; peptic or duodenal ulcer or metabolic, cardiac, malignancy diseases autoimmune and thyroid disease, as well as disorders which cause gastrointestinal bleeding, helminthic infections or malabsorption syndromes [10,60,61,62]. Further, we excluded pregnant women, women treated for abnormal uterine bleeding, in particular heavy menstrual bleeding [63], and any women involved in weight loss programs, diet restriction, or under medication known to interfere with vitamin D metabolism [64]. Participants were excluded if they had donated blood within the past three months [65], reported taking iron or vitamin D3 supplements in the past six months, used multiple micronutrient supplements, or had known iron deficiency or anemia.

The ethics committee of biomedical research at Moulay Ismail University (reference: N°1/CERB-UMI/19) approved the study protocol, and all investigations were conducted in accordance with the principles of the Declaration of Helsinki. All participants signed written informed consent. For illiterate participants, the consent form was orally explained, and a witness confirmed the participant’s understanding and agreement. Illiterate participants provided a thumbprint in place of a signature, following ethical guidelines for informed consent.

### 2.2. Measurements

#### 2.2.1. Socio-Demographic and Anthropometric Measurements

The socio-demographic covariates were collected by face-to-face questionnaire and included age, education level (Illiterate, ≤10 years (secondary–college); ≥11 years (university or higher)) and geographic localization (Urban, Rural).

Anthropometric measurements were performed with standard procedures, while participants were minimally clothed and without shoes. The weight in kilograms was measured with a digital scale (SECA^®^, Hamburg, Germany) with a precision of 0.5 kg. Height was measured with a portable stadiometer (SECA 214, Hamburg, Germany) to the nearest 0.1 cm. Body Mass Index (BMI) was calculated as weight in kilograms divided by height in meters squared and expressed in kg/m^2^. Anthropometric status was categorized using classification according to BMI as follows: Underweight: BMI < 18.5 kg/m^2^; Normal: BMI: 18.5–24.9 kg/m^2^; Overweight: BMI 25–29.9 kg/m^2^; and Obese ≥ 30 kg/m^2^ [66].

#### 2.2.2. Lifestyle Factors

##### Nutrient Intakes Estimate

Vitamin D3 dietary intakes were assessed using our validated Vitamin D-FFQ in Moroccan WRA [49]. Briefly, the VD-FFQ includes 78 items pertaining to the richest vitamin D3 food consumed in the Moroccan context. Criterion validity of the FFQ showed a high validity coefficient against seven days estimated record ρQR = 0.90 (95%CI: 0.89–0.92) [49]. Iron daily intake was estimated through 24-h dietary recall. A certified dietitian asked all women to recall all the foods and beverages consumed the day before. Photograph aids of typical Moroccan household measurements were used to assist the participant [67]. To estimate daily nutrient (vitamin D3 and iron) intake from diet, frequency and serving size for each food consumed were multiplied by the nutrient content of that food using the French CIQUAl food composition database [68] and the Nutrient Database for Dietary Studies 2015–2016 of the United States Department of Agriculture National Food (USDA), when values were missing in the French database [69].

Iron status is more dependent on the type of dietary iron (heminic and non-heminic) than on overall intake. Because of certain hemoglobin transporters [61], heminic iron is absorbed more efficiently, whereas non-heminic iron needs iron reduction prior to absorption [61]. In our estimate, the content of heme-iron was calculated based on a commonly applied assumption that heme iron is attributed to 40% of iron derived from animal products, including red meat, poultry, fish, and animal organs, and non-heme iron was calculated as the remaining portion of the total iron from all foods [70,71]. Energy intakes in Kcal/day were calculated from all participants’ 24 h recall, and nutrient estimates were adjusted for using the residual method [72]. The WHO/FAO recommended daily intakes were applied to ascertain intake adequacy of vitamin D3 and iron [73].

##### Sun Exposure Behaviors

Participants’ sun exposure habits over the previous month were assessed using our validated sun exposure score questionnaire, as described in previously published studies [49,55]. Three domains relating to sun exposure behaviors were reported and scored (on a scale of 0 to 4) (indoor sun exposure, outdoor sun exposure, including work or routine activities, recreation or leisure activities, and sun protection practices) and were then adjusted for the participants Fitzpatrick’s skin type and the strength of UVB rays in the outside weather conditions. The estimated total sun exposure score (SES) served to categorize participant exposure levels from 0 to 30. The sun exposure level was considered insufficient if SES < 17, moderate if SES = 7.5 to 15, sufficient if SES = 15 to 30, and very sufficient or high if the score was >30 [49,55].

##### Physical Activity Assessment

To assess physical activity (PA) levels, we used the short version of the International PA Questionnaire (IPAQ-SF), which is commonly applied in studies among adults aged 18–69 years [74,75]. Participants had to declare their activities during the previous week, including walking (any type of walking), moderate activities (carrying light loads, gardening, doubles tennis, and cycling at a steady pace), and vigorous activities (carrying heavy loads, aerobics, weight training, fast cycling, or jogging at 10 km/h) [75]. Each activity (walking, moderate, and vigorous activities) converted in minutes per week was multiplied by metabolic equivalent (METs) factors as follows: (daily minutes of walking × days per week with walking × 3.3 METs) + (daily minutes of moderate-intensity activity × days per week with moderate-intensity activity × 4 METs) + (daily minutes of vigorous activity × days per week with vigorous activity × 8 METs). The score was categorized as low intensity (<600 MET-min/week), moderate PA (at least 600 MET-min/week), and intense/vigorous PA (at least 3000 MET-min/week) [74].

All questionnaires, including the IPAQ, were administered through face-to-face interviews conducted by a trained dietitian and nurses. This approach ensured full comprehension and accurate responses from both literate and illiterate participants.

##### Laboratory Assessment

Fasting venous blood samples (10 mL) were collected from each participant and analyzed on the same day. Participants were instructed to fast for eight to twelve hours before sample collection, following laboratory norms. Serum 25(OH)D and concentrations were performed with a one-step electrochemiluminescence immunoassay according to the manufacturer’s instructions. All chemical analyses were conducted using the Abbott Architect ci4100 Chemistry Analyzer COBAS E411 analyzer Abbott ARCHITECT ci4100 Chemistry Analyzer (Abbott Laboratories, Abbott Park, IL, USA) and Roche COBAS e 411 Analyzer (Roche Diagnostics, Basel, Switzerland). Blood count (BC) analysis was performed using automates (BC-5380). Unless otherwise specified, we use laboratory references to express the proportion of each biomarker’s category concentration (low or high).

##### Biomarkers Cut-Off Points Definition

Anemia and severity of anemia were defined according to the WHO criteria for anemia: Hb level < 12 g/dL, for the severity of anemia; mild anemia: HB: 11–11.9; moderate anemia: HB: 11–8-10.9; severe anemia HB < 8 g/dL [8]. The diagnostic criteria for ID were serum ferritin (SF) concentration < 15 μg/L in the absence of inflammation (CRP < 5 mg/L) [8,10,76]. IDA was diagnosed by the criteria for iron deficiency in addition to lower hemoglobin of less than 12 g/dL [8,10,76].

VDD was defined as serum 25(OH)D levels < 20 ng/mL [36,77,78], a threshold associated with the prevention of impaired intestinal calcium absorption and for maintaining skeletal and overall health [78,79]. Severe VDD was defined as 25(OH)D < 12 ng/mL [41,42,43], whereas vitamin D insufficiency was defined as levels between 20 and 30 ng/mL [36,78]. Women with serum 25(OH)D > 30 ng/mL concentrations were considered to have sufficient levels [36,78].

Furthermore, the Modification of Diet in Renal Disease (MDRD) formula was used to assess normal renal function [80]. Only datasets with an estimated glomerular filtration rate (eGFR) greater than 60 mL/min/1.73 m^2^ were included in the final analysis [80].

### 2.3. Statistical Analysis

The normality of the continuous data was evaluated using Kolmogorov–Smirnov and Shapiro–Wilk tests (n > 50), respectively. All variables were not normally distributed; therefore, only nonparametric tests were applied. Descriptive analysis was performed, and data were expressed as median with interquartile range (IQR) for continuous variables and in frequencies and percentages for categorical variables.

Participants were classified into sub-groups according to vitamin D and anemia status. Kruskal–Wallis tests were used to compare groups of hypothesized risk factors (independent variables) according to vitamin D or anemia status sub-groups, while chi-squared or Fisher’s exact test was used to measure the difference in frequency for categorical variables.

Pearson correlation coefficient and linear regression analysis were used to evaluate the relationship between continuous variables (25(OH)D and iron/anemia markers, which were log-transformed for scatter plots and regression analyses.

To investigate the associations between VDD, ID, IDA, and all-cause anemia, we utilized a combination of logistic regression and Receiver Operating Characteristic (ROC) curve analysis.

#### 2.3.1. Logistic Regression

Logistic regression models were employed to estimate the odds ratios (OR) and corresponding 95% confidence intervals (CI) for the associations between VDD and the outcomes of ID, IDA, and anemia. To ensure the accuracy of the logistic regression models, the collinearity between potential predictors was checked using the Variance Inflation Factor (VIF), with a threshold of VIF < 10 indicating acceptable collinearity [81]. Stepwise regression analysis was then applied to select significant predictors of the outcomes to be included in the multivariate models. The best model was retained based on the Akaike Information Criterion (AIC), ensuring the most parsimonious model with the best fit. The significance of the predictors was assessed using z-values and *p*-values [81].

#### 2.3.2. ROC Analysis

To assess the discriminative power of VDD in the logistic models, ROC curve analysis was conducted [82]. The ROC curves for both the full model and VDD-specific model were generated, providing visual representations of the sensitivity and specificity of the models. The area under the curve (AUC) was calculated to quantify the overall accuracy of the models in distinguishing between anemia, IDA, ID cases, and non-cases. Commonly used values for AUC interpretation are 0.90–1.00: Excellent; 0.80–0.89: Good; 0.70–0.79: Fair; 0.60–0.69: Poor; and 0.50–0.59. Sensitivity and specificity above 0.70 are regarded as acceptable [82].

All statistical analyses were performed in R version 3.4.2 (28 September 2017). A *p*-value of less than 0.05 was considered statistically significant for this study.

## 3. Results

Baseline characteristics of the study participants overall and by anemia status are presented in Table 1. Anemia was present in 23.3% of women participants, and 76.6% were healthy. There was no statistically significant difference in terms of most socio-demographics between the healthy and anemic groups. However, urban women exhibited a significantly higher prevalence of anemia (55.6%) compared to their rural counterparts (44.4%) (*p* = 0.025).

The median BMI indicated a higher prevalence of overweight and obesity among our study participants, with 61.3% falling into this category (38.0% overweight and 23.3% obese). Anemia was associated with a higher median (IQR) BMI (*p* = 0.001) and showed a significantly higher prevalence rate (72.2%, *p* = 0.024) in the overweight and obese groups (39.8% and 32.4%, respectively).

The PAC score median (IQR) was determined to be 1647 MET-min/week. Among the women participants, 80.7% exhibited a moderate activity level, 11.5% had a low PAC level, and 7.8% had a high PAC level. A lower median (IQR) PAC level was observed in the anemic group (*p* < 0.0001).

Regarding dietary intakes, our results showed that most women, 81.2% and 100%, reported inadequate intake of vitamin D and iron, falling below the recommended values established by WHO and FAO. The anemic group displayed a significantly lower intake of vitamin D compared to the healthy group (*p* = 0.035). Non-heme iron consumption was moreover higher in the healthy participants (*p* = 0.012). Further, more than half of our participants (53.3%) reported insufficient to moderate sun exposure scores, with the highest proportion (55.6%) observed in the anemic women (*p* = 0.034).

Data on biochemical parameters according to the participants’ 25(OH)D (ng/mL) cut-off points are described in Table 2. The median (IQR) 25(OH)D level for the overall study population was 14.4 (10.8) ng/mL within 463 women participants included in our analysis. VDD showed the highest rate with 72.1%, of which approximately one-third (29.7%) had a severe VDD. Vitamin D insufficiency affected 17.1%, whereas only 10.2% of our participants had sufficient 25(OH)D3 levels.

A pattern of significant increase from the deficiency to insufficiency range was observed in almost all blood count markers, including hemoglobin (*p* < 0.001), erythrocytes (*p* = 0.005), hematocrit(*p* < 0.001), MCV(*p* < 0.001), MCH(*p* < 0.001), and MCHC (*p* < 0.001). WBC count seems to be inversely correlated with serum 25(OH)D concentration, as higher counts were observed in the VDD range, but this was not statistically significant (*p* = 0.643).

Higher ferritin concentrations were observed in participants with sufficient levels of vitamin D, showing an increasing trend (*p* = 0.002). However, the distribution of iron concentration levels did not differ significantly across vitamin D status ranges (*p* = 0.604). Related to renal function parameters, none of our participants had chronic kidney disease as the computed rate of eGFR < 60 mL/min/1.73 m^2^ was sited to 0. However, participants eGFR (mL/min/1.73 m^2^) exhibits a substantial increase (*p* = 0.045) as the 25(OH)D levels rise while plasma creatinine remains stable. When considering the presence of inflammation (defined by CRP > 5), 21.8% of all participants had inflammation, with no significant difference in this proportion across 25(OH)D levels.

**Figure 2:**
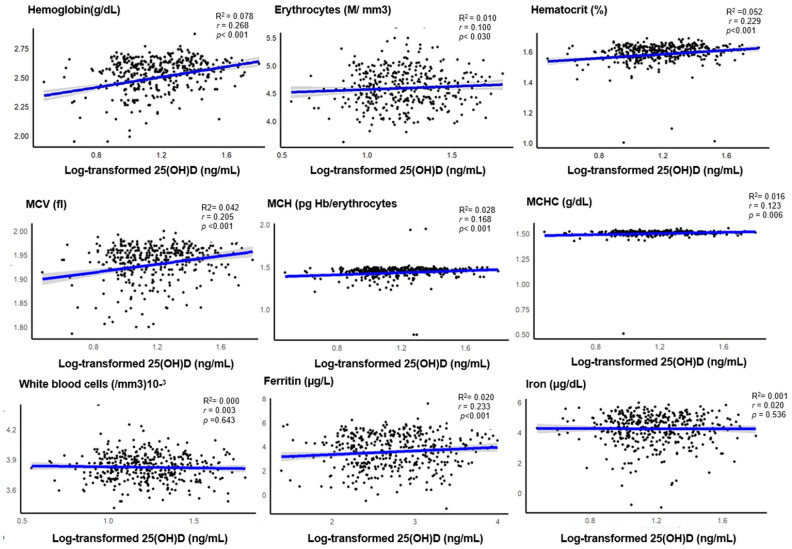
Scatter and regression plots of anemia indices against log 25(OH)D3 levels. Abbreviations: 25(OH)D3: 25-hydroxyvitamin D, MCV: Mean corpuscular volume, MCH: mean corpuscular hemoglobin, MCHC: mean corpuscular hemoglobin concentration, r: Pearson correlation coefficient, Magnitude of the correlation: Negligible (r: 0.00–0.10), weak (r: 0.10–0.39), moderate (r: 0.40–0.69), strong (r: 0.70- 0.89), very strong (r: 0.90–1.00) [83]. *p* < 0.05.

As illustrated in Figure 2, all blood count parameters, including hemoglobin, hematocrit, erythrocytes (red blood cells), MCV, MCH, and MCHC, were positively related to circulating 25(OH)D, except for white blood count, which showed no significant association with vitamin D levels. Additionally, a significant association was found between 25(OH)D and ferritin, whereas iron concentration was not related to 25(OH)D concentration. The magnitude of these relationships was weak for all indices (all r: 0.10–0.39, *p* < 0.05) [83].

Table 3 presents a detailed distribution of anemia and iron status according to the levels of 25(OH)D3 serum concentrations in the study participants.

The distribution of anemia varied significantly across different 25(OH)D levels (*p* < 0.001). The highest prevalence of anemia (93.5%) was observed in participants with 25(OH)D levels indicative of VDD (25(OH)D < 20 ng/mL), compared to a proportion of 65.6% in the non-anemic women. However, anemia severity distribution was not statistically different across participants’ vitamin D status.

In addition, of the study participants, 27.6% were found to have ID. The majority (82.6%) of these women fell within the VDD range. The variations in ID rates across different 25(OH)D statuses were statistically significant (*p* = 0.001). The rate of IDA within the anemic group was 50% (54 out of 108), while among all women participants, 11.7% (54 of 463) presented IDA. Of these, a considerable proportion (90.7%) presented concurrent VDD (*p* = 0.003), compared to a proportion of 69.7% in the non-anemic group (*p* = 0.003).

The logistic regression analysis results, as presented in Table 4, indicate a significant association between VDD and anemia. Women with VDD had an odds ratio (OR) of 8.79 (95% CI: 4.06, 23.00; *p* < 0.001) for anemia, suggesting a substantially higher likelihood of developing anemia compared to those with insufficient or sufficient vitamin D levels. After adjusting for potential confounders, the OR for the association between VDD and anemia was slightly reduced to 7.17 (95% CI: 3.19, 19.28) but remained highly significant (*p* < 0.001).

Similar trends were observed regarding the impact of VDD on iron status. Women with VDD were found to be twice as likely to present with ID and over four times more likely to have IDA compared to those without VDD. The unadjusted OR for ID was 2.12 (95% CI: 1.28, 3.50; *p* = 0.003), which, after adjustment for confounders, slightly increased to 2.20 (95% CI: 1.32, 3.77; *p* = 0.007). For IDA, the unadjusted OR was 4.16 (95% CI: 1.62, 10.71; *p* = 0.003), and the adjusted OR was 4.10 (95% CI: 1.73, 12.08; *p* = 0.004).

**Figure 3:**
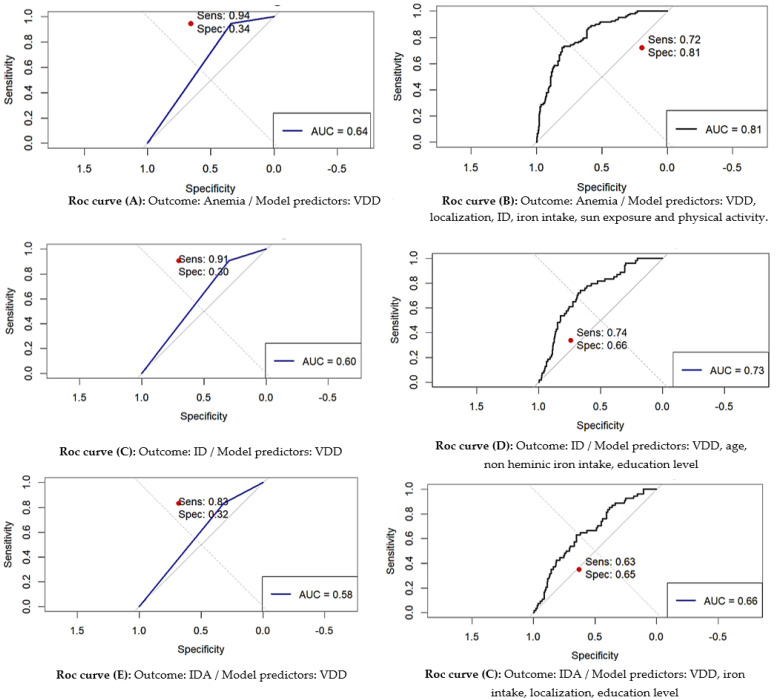
ROC curves of VDD ability in predicting anemia and iron status. Abbreviations: VDD: Vitamin D deficiency, ID: iron deficiency, IDA: Iron deficiency anemia, AUC: Area under the curve, Sens: sensitivity, Spec: specificity. Statistical significance, *p* < 0.05.

Summary of ROC analysis results are presented in Table 5 and Figure 3. The AUC for VDD predicting general anemia and specific IDA were 0.643 (*p* < 0.001) and 0.603 (*p* < 0.001), respectively, indicating moderate discriminant ability. Higher sensitivity values in identifying cases were observed (0.944 for anemia and 0.833 for IDA), with Youden indexes indicating a moderate balance between sensitivity and specificity for VDD in predicting anemia and IDA (0.285 and 0.206, respectively).

The inclusion of covariates in the model, such as ID, geographic localization, dietary iron intake, sun exposure, and physical activity level, likely contributed to the higher accuracy and predictive power for anemia (AUC: 0.813 (95% CI: 0.768–0.855), *p* < 0.001, optimal sensitivity: 0.722, optimal specificity: 0.806, and Youden Index: 0.528).

Similarly, the adjustment for covariates contributed to the balanced sensitivity and specificity and more moderate discrimination ability of VDD in predicting IDA (AUC: 0.712 (95% CI: 0.643–0.776), *p* < 0.001, optimal sensitivity: 0.741, optimal specificity: 0.648, and Youden Index: 0.389).

Nevertheless, the accuracy of VDD in predicting ID appears low, with an AUC of 0.575 (*p* < 0.001), although moderate sensitivity was observed (0.833). Accounting for participants’ demographic characteristics, including age and education level, in addition to dietary intake of non-heminic iron, improved the accuracy of VDD to detect anemia cases with an acceptable AUC of around 0.654, specificity of 0.634, and sensitivity of 0.648.

## 4. Discussion

Vitamin D and iron deficiencies and the resulting anemia remain widespread among Moroccan WRA despite existing health management efforts. This cross-sectional study examined the interplay between VDD, iron status, and anemia in 463 WRA aged 18–49 from Meknes, Morocco.

The prevalence of anemia in our study (23.3%) was lower than the national rate of 34.4% [51] and findings from studies in eastern (Essaouira, 41%) [84] and northern Morocco (El M’diq-Fnideq, 44%) [85]. This discrepancy may be due to differences in sampling methods, inclusion criteria (as the referenced studies included both pregnant and non-pregnant women), and hemoglobin measurement techniques, such as the Haemoglobinometer used in the 2019–2020 national nutritional survey. However, the prevalence rates of ID and IDA in our study were consistent with national reports [51], highlighting the ongoing challenges in addressing nutritional deficiencies among premenopausal women.

We observed a high prevalence of VDD (72.1%) in our study, with approximately one-third of participants experiencing severe VDD, consistent with national rates [51]. In a study by Dadda [48] conducted in the arid Draa-Tafilalet region, vitamin D status was even lower, with over half of female participants showing 25(OH)D levels below 10 ng/mL, highlighting a significant health concern. Beyond skeletal health implications [14], VDD in these women may also affect cardiovascular health [14,54]. A previous analysis of this study’s subset (n = 300) by Lhilali [50] found that vitamin D-deficient women had a 3.5 to 5-fold increased risk of elevated atherogenic indices, particularly non-HDL cholesterol, and hypertriglyceridemia, indicating heightened cardiovascular susceptibility. These findings imply that young women with low 25(OH)D levels may face a heightened risk of early cardiovascular issues, emphasizing the need to address vitamin D deficiency as a preventive measure.

Studies on the impact of VDD on iron metabolism and anemia have shown inconsistent results [25,27,28,29,30,31,86,87,88]. One proposed mechanism is the suppression of hepcidin, a liver hormone regulating iron release [21]. Vitamin D, particularly its active form (1α,25(OH)_2_D_3_), can downregulate hepcidin expression via direct interaction with the vitamin D receptor on the HAMP gene and its suppression of pro-inflammatory cytokines (e.g., IL-6), potentially enhancing iron availability for erythropoiesis and hemoglobin synthesis [22]. However, some studies do not show a significant effect of vitamin D on hepcidin levels, suggesting further research is needed to confirm these pathways across different populations [89,90].

Calcitriol may also support erythropoiesis by directly stimulating erythroid progenitors alongside erythropoietin (EPO), reducing the need for higher EPO doses, as seen in kidney disease [18,19] and pregnancy [91]. Elevated vitamin D levels in the bone marrow, compared to serum, support this role in hematopoiesis, possibly in an autocrine manner [20]. Additionally, a recent study by Zhao [92] in animals showed that vitamin D supports iron homeostasis by regulating divalent metal transporter 1 (DMT1), critical for iron uptake from the intestine into cells. This potential action may influence iron uptake across key metabolic compartments, including erythroid cells, which rely on DMT1 for transferrin-dependent iron transport [93].

Our analysis revealed weak but significant correlations between vitamin D levels and biomarkers of anemia and iron status (e.g., hemoglobin, RBC, hematocrit, MCV, MCH, MCHC, and ferritin; r: 0.10–0.39, *p* < 0.05). This weak association may be due to the low vitamin D concentrations in our participants (median (IQR): 14.4 (10.8) ng/mL), though markers tended to increase as vitamin D levels moved from deficient to insufficient (All *p* < 0.01). This trend aligns with vitamin D’s potential role in enhancing erythropoiesis, iron storage, and mobilization. Given that inflammation can alter ferritin via hepcidin upregulation, vitamin D may help normalize ferritin and improve iron availability by reducing inflammation [94].

A significant interaction between VDD, ID, and anemia emerged from our analysis, with 93.5% of anemic women, 90.7% of those with IDA, and 82.6% of those with ID also experiencing concomitant VDD. Notably, VDD was a strong independent predictor of anemia (OR: 7.17, 95% CI: 3.19–19.28), ID (OR: 2.20, 95% CI: 1.32–3.77), and IDA (OR: 4.10, 95% CI: 1.73–12.08). These findings suggest that maintaining adequate vitamin D levels (≥20 ng/mL) may be critical for managing iron deficiency and anemia in Moroccan WRA, potentially offering a preventive approach aligned with public health interventions.

The current study results resonate with prior research among both healthy and patient populations of premenopausal women [25,30,87,88,95,96]. Particularly, a large cohort study conducted by Zhang [97] involved a US adult population (n = 29,933, with 50.5% women). The study found that increases in RBC count and hemoglobin levels were correlated with higher serum 25(OH)D levels, specifically within the insufficiency range of 23.8–28.1 ng/mL (59.7–70.3 nmol/L), in addition to a reduced incidence of anemia [97]. The study by Seong [88] in a nationally representative survey involving 16,060 Korean adults, where severe VDD (<15.0 ng/mL) was significantly associated with lower hemoglobin, hematocrit, iron, and ferritin concentrations in premenopausal women. The risk of anemia in these women was significantly higher (OR = 1.293, 95% CI: 1.105–1.513, *p* < 0.001). At the same citing, Shin [87] previously reported a VDD prevalence of 94.9% in premenopausal women, with a significant odds ratio for anemia in the lowest vitamin D quartile (Q4, ≤11.92 ng/mL) at OR = 1.821 (95% CI: 1.240–2.673), *p* = 0.009. Likewise, a meta-analysis by Liu [27] of five studies involving adult participants concluded that VDD was associated with an increased incidence of anemia in both genders (OR = 2.33; 95% CI = 1.43–3.80).

However, it is important to note the conflicting results from other studies. Smith [98] reported in a US cohort of adult men and women a correlation between low serum 25(OH)D levels and increased odds of anemia in Black individuals but not in Whites, particularly associating it with anemia of inflammation without impacting iron status [98]. Conversely, Malczewska-Lenczowska [25] observed that VDD (<30 ng/mL) was associated with both storage and transport iron pools such in lower ferritin levels, higher Total Iron-Binding Capacity (TIBC), and elevated soluble transferrin receptor (sTfR) levels, indicating a prevalent pattern of ID in athletic female (OR: 1.75, 95% CI: 1.02–2.99, *p* = 0.040; OR: 4.6, 95% CI: 1.81–11.65, *p* = 0.001) [25]. Similarly, Lavoie [99] found that 25(OH)D levels were positively associated with serum ferritin (β: 12, 95% CI: 0.01–0.22, *p* < 0.05) and inversely associated with IDA (β: 0.57, 95% CI: 0.38–0.84, *p* < 0.01), though no association with hemoglobin levels was observed in both referred studies [99].

In Arab populations, El-Adawy [100] found no significant correlation between vitamin D levels and iron or hemoglobin indices in Egyptian female adolescents, while a large retrospective Saudi study by Alfhili [101] also found no association between VDD and anemia (OR: 0.90, 95% CI: 0.64–1.27, *p* = 0.578). However, hypocalcemia was significantly associated with a higher anemia risk across all age groups (OR: 3.32, 95% CI: 2.27–4.86, *p* < 0.0001) [101].

These discrepancies suggest that vitamin D’s role in mitigating anemia may vary by geography, demographics, and anemia type, potentially influenced by ethnic factors [24,95,98]. Morocco’s diverse genetic background, including African, Berber, and Arab ancestry, may influence iron absorption and anemia etiology [102,103] in addition to the VDD metabolic pathway [104]. This diversity may partly explain the stronger associations observed in our study compared to other populations.

Although our study focuses on adult women, other local studies on children report a higher prevalence of hereditary hemoglobinopathies, such as Thalassemia, in north Morrocco [105,106]. North Moroccan sickle cell disease patients have shown a low frequency of the XmnI polymorphism associated with higher fetal hemoglobin levels and potentially milder disease severity compared to haplotypes observed in Senegalese and Arab Indian populations [107]. This reflects genetic variability in determinants of anemia within the Moroccan population.

Research shows that African American populations have lower hemoglobin concentrations than European or South Asian populations despite similar iron intake and status [103]. African and Moroccan women also present the lowest vitamin D levels, even with abundant sunlight [38,108], with studies in Morocco showing particularly low levels in summer [42] and in drier regions [43,48].

Dark skin, which limits vitamin D synthesis from UV light, is a key factor contributing to VDD in Moroccan and African populations [38,48]. Individuals with darker skin may require up to 10 times more UVB exposure to achieve similar increases in blood vitamin D3 levels as those with lighter skin [109]. Additionally, Pigmented skin tends to be associated with lower CYP27B1 activity (which converts 25-hydroxyvitamin D to its active form) and higher CYP24A1 activity (which breaks down 25-hydroxyvitamin D), likely contributing to reduced circulating [104]. This enzymatic profile affects overall vitamin D availability in darker-skinned populations, suggesting that vitamin D metabolism may be partly influenced by genetic ancestry [104].

Interactions between vitamin D and iron may be influenced by baseline health conditions [24,26,30,95,99]. Greenwood’s study [95] in Auckland found no association between serum 25(OH)D and iron or hemoglobin status across ethnic groups. However, South Asian women paradoxically showed higher hepcidin with elevated 25(OH)D levels, possibly due to differences in body fat and inflammatory cytokine levels-IL-6. Similarly, Soepnel [30] found no correlation between anemia or ID and vitamin D status among South African women but noted a negative association between fat mass index and 25(OH)D levels. In our study, higher BMI was significantly linked to anemia (*p* = 0.001), with 72.2% of anemic women being overweight or obese, compared to 58.1% of non-anemic women.

Obesity-related factors, such as chronic inflammation and elevated hepcidin, contribute to ID and anemia [110], while vitamin D sequestration in adipose tissue may explain the high VDD rates observed [111]. Our logistic regression showed that obese participants had a 24% higher risk of VDD than women with normal BMI (Table S1), consistent with findings in Moroccan studies [54]. Nationally, anemia coexists with overweight and obesity in 62.8% of Moroccan WRA (33.1% obese, 29.7% overweight), with severe VDD rates highest in obese women (36.9%) compared to those with normal weight (26.4%) or overweight (30.7%) [51]. These findings suggest that obesity contributes to the burden of both anemia and VDD, underscoring the need for targeted interventions to address the combined risks associated with high adiposity in WRA.

Environmental factors, particularly sunlight exposure, are crucial to vitamin D status [12]. Emerging studies suggest that ultraviolet radiation might affect blood gene expression related to inflammatory responses, cytokine regulation, and cellular transport, potentially impacting hepcidin and iron metabolism beyond its direct effects on vitamin D synthesis and plasma 25(OH)D levels [112]. Lifestyle choices, such as wearing protective clothing, staying indoors, and using sunscreen, are often associated with inadequate sun exposure and VDD in women [42,48,55].

Sun exposure duration of less than 20 min per day is associated with the high rate of severe VDD among Moroccan WRA [51]. Our bivariate analysis shows that women with insufficient to moderate sun exposure have a 59% higher likelihood of anemia (*p* = 0.034), though this association was not significant in the multivariate model when VDD and other factors were included. Women with insufficient sun exposure are also 2.4 times more likely to have VDD than those with sufficient exposure (*p* < 0.001) (Table S1). This strong association suggests that the link between sun exposure and anemia may be primarily mediated by vitamin D levels, partially explaining the high prevalence of VDD in the anemic group.

The association between low physical activity and anemia observed in our sample is further supported by evidence showing that anemia can impair aerobic capacity, as indicated by decreased maximal oxygen consumption (VO2max), which affects physical fitness and endurance [113]. This could suggest a cyclical relationship where sedentary behavior contributes to anemia, which in turn accented fatigue and reduces physical activity levels. However, whether sedentary behavior is primarily a cause or a consequence of altered iron status and hemoglobin levels remains to be fully elucidated [114].

Furthermore, urban residency emerged as an independent risk factor for anemia in our study. Urban living may reduce opportunities for outdoor exposure to sunlight, thereby decreasing UVB availability for vitamin D production [115]. In a study by Lhilali [50], rural women had higher median sun exposure scores due to greater indoor sunlight access inside their homes and yards compared to urban women living in apartments with limited direct sunlight. Other representative study reports higher rates of severe VDD among urban Moroccan WRA. With urban women comprising 64.6% of our sample, reduced UV sun exposure may further increase VDD and its associated anemia risk.

While there is debate over vitamin D content in foods and required intake [13,116], dietary sources and body stores support adequate levels when sun exposure is limited [116,117]. In Morocco, studies report low vitamin D intake among WRA [49,118] and up to 2 times the risk of VDD associated with low calcium intake [42]. Vitamin D-fortified products (e.g., edible oil) are consumed by 57.5% of WRA [51], contributing to less than 5% of the recommended daily intake [49].

Our analysis showed that only 18.8% of our participants met the recommended daily intake of 5 µg/day. However, a clear dose-response relationship was observed, with the highest quartile of vitamin D intake (Q4: 4.41–16 μg/day) significantly associated with higher 25(OH)D concentrations compared to Q1(0.2–1.67 µg/d) after adjusting for cofactors, including ferritin and hemoglobin (β (SE): 0.16 (0.06), *p* = 0.008) (Table S2). Anemic women were consuming significantly less iron and vitamin D than their non-anemic counterparts (*p* = 0.011 and *p* = 0.031, respectively). These results are notable as they suggest that even modest increases in dietary vitamin D intake may improve serum 25(OH)D concentrations and hemoglobin status, potentially contributing to deficiency risks associated with low sun exposure in this population.

In the Rabat-Sale-Kenitra region, Barich [119] found inadequate iron intake (10.02 mg/day on average) among 542 women (ages 19–49. Consistent with these findings, we found significantly low iron intake, with 95.61% from non-heme and only 4.39% from bioavailable heme iron, insufficient to offset physiological losses and contributing to anemia. Anemic women consumed significantly less iron than non-anemic women (*p* = 0.011), though heme iron intake was low across both groups (*p* = 0.051). Dietary iron intake provided minimal protection against anemia and IDA (β(SE): −0.05(0.02), *p* = 0.041; β (SE): −0.08(0.03), *p* = 0.030), with non-heme iron intake showing a negative association with ID (β(SE): −0.05(0.02), *p* = 0.037). The dietary habits of Moroccan WRA, relying on vegetable sources, non-heme iron-rich legumes, vegetables, and cereals, which are also low in vitamin D [51,84,120], may explain these results.

Broader dietary fortification and public health efforts should promote vitamin D and iron-rich animal sources. Effective interventions must prioritize dietary diversity to ensure adequate micronutrient intake in WRA and enhanced iron absorption, with further studies refining supplementation based on sun exposure, season, and adiposity. Clinical trials are needed to assess the role of vitamin D intake in managing iron deficiency and anemia.

The ROC analysis showed that VDD had moderate discriminative power for diagnosing general anemia (AUC: 0.643) and IDA (AUC: 0.603), with high sensitivity but low specificity (Youden index: 0.285 for anemia, 0.206 for IDA). The low specificity indicates that while VDD is useful for identifying individuals at risk of anemia, it is not sufficient as a standalone diagnostic marker due to its inability to reliably distinguish between anemia types or other causes of poor iron status. This limitation is further demonstrated by the particularly weak performance of VDD in predicting iron deficiency alone (AUC: 0.575, sensitivity: 0.833). Thus, for more accurate clinical assessment and intervention planning, VDD should be evaluated in conjunction with a comprehensive profile of other diagnostic markers and patient-specific risk factors (e.g., sun exposure, dietary intake, physical activity, BMI, and socio-demographic factors), particularly in WRA, who are vulnerable to multiple nutritional deficiencies.

Finally, methodological differences in defining VDD thresholds contribute to variability in findings, potentially leading to overestimated VDD rates in Moroccan women and the MENA region. International guidelines set 25(OH)D levels ≥ 20 ng/mL for bone health, with <20 ng/mL associated with increased PTH and bone loss [121]. Moroccan studies present varied results. Alali [42] reported 91% insufficiency among older women, with weak correlations to bone markers; El Maghraoui [45] linked severe deficiency (<10 ng/mL) to vertebral fractures in menopausal women; Ibn Yaacoub [44] found a mean 25(OH)D of 10.88 ng/mL among women with systemic sclerosis, significantly lower than controls (*p* = 0.001). In a broader sample, Mehdad [53] reported 74.4% VDD in women aged 18–65, with significant correlations to BMI and PTH in overweight women.

These findings are heterogeneous, with a focus on older and postmenopausal women. Further research across age groups is needed to establish appropriate VDD thresholds for Morocco and enable accurate comparisons with international reports.

This study offers valuable insights into vitamin D intake and sun exposure behavior among Moroccan women, utilizing validated questionnaires that enhance the quality of nutrient intake data. By adjusting for a wide range of confounders, including BMI, lifestyle factors, and socioeconomic characteristics, our analysis provides a robust examination of the associations between vitamin D status, iron levels, and anemia. As the first study to examine these relationships specifically in Moroccan women, our findings add valuable insights into vitamin D and iron status within this population, contributing to a more comprehensive understanding in this context.

However, some limitations exist. The single 24-h recall for iron intake may not accurately reflect participants’ usual intake, and the cross-sectional design restricts causal inferences between VDD, iron status, and anemia risk. Direct measurements of hepcidin or pro-inflammatory cytokines were not included, limiting our exploration of whether the associations between VDD and iron status are mediated by inflammatory pathways. Although we observed no correlation between general inflammatory markers (e.g., WBC, CRP) and vitamin D or iron levels, this does not rule out subtle inflammatory mechanisms that could influence these associations.

Further, we did not assess other nutritional causes of anemia, such as B12 and folate levels, focusing only on iron deficiency. To minimize confounding, we excluded participants with chronic diseases, infections, and documented causes of anemia. Our sample (n = 463) from a single region (Meknes) may also limit the generalizability of findings to the broader population of Moroccan WRA.

Future longitudinal studies with larger, more diverse samples and the inclusion of biomarkers like hepcidin and transferrin receptors are needed to examine the temporal dynamics of these associations and to support more targeted prevention and management strategies.

## 5. Conclusions

In summary, our study adds to the growing body of evidence that VDD significantly impacts iron homeostasis in premenopausal women, increasing the risk of anemia and poor iron status among Moroccan WRA. Addressing VDD in this at-risk group could not only support bone health but also enhance iron metabolism, potentially preventing iron deficiency and anemia. Clinically, recognizing that VDD and iron deficiency are interrelated and often coexist in anemic WRA is crucial. Our findings suggest that routine assessment of VDD as part of a comprehensive evaluation of anemic women could offer valuable insights into the multifactorial causes of anemia and IDA. This integrated approach may improve diagnostic precision and lead to more effective, individualized treatment strategies. Further clinical trials are sought to determine the efficacy of vitamin D supplementation as an adjunct therapy in managing ID and anemia.

In the current context, there is also an urgent need for targeted public health interventions and awareness campaigns to promote adequate dietary intake, sun exposure, and overall healthy lifestyle choices among young Moroccan women. These efforts could play a crucial role in reducing the dual burden of VDD and anemia in this vulnerable population.

## Figures and Tables

**Table 1:** Characteristics of study participants by serum 25(OH)D3 and anemia status.

Characteristics	All Participants (n = 463)	Anemic (n = 108)	Non Anemic (n = 355)	*p*-Value ^1^
	Median (IQR) or n (%)			
**Anemia status (HB < 12 g/dL)**	463 (100)	108(23.3)	355(76.6)	<0.001
**Socio-demographic characteristics:**				
**Age, years**	29.0 (11.0)	28.0 (10.0)	29.0 (11.0)	0.639
**Education**				
Illiterate	84 (18.1)	20 (18.5)	64 (18.0)	
≤10 years (secondary, college)	222 (47.9)	56 (51.9)	166 (46.8)	
≥11 years (university or higher)	157 (33.9)	32 (29.6)	125 (35.2)	0.259
**Localization**				
Urban	299 (64.6)	60 (55.6)	239 (67.3)	
Rural	164 (35.4)	48 (44.4)	116 (32.7)	0.025
**Anthropometric measures**				
BMI, kg/m^2^	26.5 (6.4)	27.6 (7.0)	25.8 (6.9)	0.001
BMI classification				
Underweight (<18 kg/m^2^)	6 (1.3)	1 (0.9)	5 (1.4)	
Normal (<25 kg/m^2^)	173 (37.4)	29 (26.9)	144 (40.6)	0.024
Overweight (25 to <30 kg/m^2^)	176 (38.0)	43 (39.8)	133 (37.5)	
Obesity (≥30 kg/m^2^)	108 (23.3)	35 (32.4)	73 (20.6)	
**Lifestyle factors;**				
**PAC score, MET min/week**	1597.6 (1892.4)	1358.4 (1178.8)	1778.2 (1765.0)	<0.0001
PAC levels (%)				<0.0001
Low	53 (11.5)	29 (12.3)	24 (6.8)	
Moderate	373 (80.7)	75 (86.4)	298 (83.9)	
High	36 (7.8)	3 (2.8)	33 (9.3)	
**Dietary intake**				
Energy, kcal/day	1678.4 (651.3)	1748.1 (758.3)	1652.6 (596.8)	0.295
Vitamin D intake, µg/day	2.7 (2.74)	2.3 (2.3)	2.8 (2.76)	0.035
Vitamin D intake categories				
Low (<5 µg/day)	376 (81.2)	91 (84.3)	285 (80.3)	
Adequate (≥5 µg/day)	87 (18.8)	17 (15.7)	70 (19.7)	0.352
Iron intake, mg/day	11.4 (6.1)	9.2 (6.4)	11.5 (5.1)	0.011
Iron intake categories				
Low (<58.8 mg/day)	463 (100)	108 (23.3)	355 (76.7)	-
Adequate (≥58.8 mg/day)	0 (0.0)	0 (0.0)	0 (0.0)	
Heminic iron intake, mg/day	0.5 (0.2)	0.4 (0.3)	0.5 (0.2)	0.051
Non-Heminic iron intake, mg/day	11.0 (5.3)	8.4 (6.5)	10.1 (5.1)	0.012
**Sun exposure behaviors**				
Sunscore (SES), median (IQR)	15.5 (8.5)	14.4 (6.9)	15.9 (10.5)	0.060
Sunscore categories (%)				
Insufficient–Moderate	247 (53.3)	60 (55.6)	156 (43.9)	0.034
Sufficient–High	216 (46.7)	48 (44.4)	199 (56.1)	

Abbreviations: IQR: interquartile range, PAC: physical activity, SES: sun exposure score. ^1^ Kruskal–Wallis test was used to compare continuous variables, and χ^2^ test or Fisher’s exact test was used to compare qualitative variables. Significant level, *p* < 0.05.

**Table 2:** Distribution of participants’ biochemical parameters in the study sample by serum concentration of 25(OH)D3 (ng/mL) cut-off points.

Parameters	All Participants (n = 463)	25(OH)D (ng/mL) Cut-Off Points			*p*-Value ^1^
		20 (n = 334)	20–30 (n = 82)	>30 (n = 47)	
	Median (IQR) or n (%)				
**25(OH)D (ng/mL)**	14.4 (10.8)	11.7 (5.6)	24.3 (4.7)	34.9 (12.2)	<0.001
**Cut-off points,**					
<12 ng/mL	99 (29.7)				
12–20 ng/mL	334 (72.1)				
20–30 ng/mL	82 (17.1)				
>30 ng/mL	47 (10.2)				
**BC parameters:**					
Hemoglobin (g/dL)	12.8 (2.2)	12.2 (3.0)	13.4 (1.5)	13.1 (1.4)	<0.001
Erythrocytes (M/ mm^3^)	4.5 (0.5)	4.4 (0.5)	4.6 (0.4)	4.5 (0.4)	0.005
Hematocrit (%)	40.0 (5.7)	38.1 (6.8)	41.5 (5.4)	40.1 (3.8)	<0.001
MCV (fl)	88.0 (8.0)	86.3 (9.7)	89.2 (6.2)	90.0 (7.0)	<0.001
MCH (pg Hb/erythrocytes)	28.0 (3.4)	27.3 (4.8)	29.0 (2.1)	29.0 (2.0)	<0.001
MCHC (g/dL)	32.1 (1.4)	31.7 (2.0)	32.3 (1.2)	32.2 (1.0)	<0.001
White blood cells (/mm^3^) 10^−3^	2.1 (0.07)	6.7 (2.6)	6.4 (3.1)	6.6 (2.8)	0.643
**Iron markers:**					
Ferritin (µg/L)	13.4 (20.8)	11.4 (15.5)	14.8 (29.4)	32.8 (44.9)	0.002
Iron (μg/dL)	55.5 (50.5)	51.1 (49.8)	53.0 (47.8)	77.3 (60.6)	0.604
**Renal function:**					
Plasma creatinine (mg/dL)	0.7 (0.1)	0.6 (0.1)	0.7 (0.1)	0.7 (0.1)	0.231
eGFR (mL/min/1.73 m^2^)	196.9 (96.16)	184.5 (93.7)	215.4 (94.0)	204.8 (86.3)	0.010
**Inflammatory marker:**					
CRP (mg/dL)	3.75 (4.57)	3.8 (4.3)	3.1 (4.9)	4.1 (6.7)	0.366
**Inflammation present (CRP > 5):**					
Yes	101 (21.8)	81 (24.3)	12 (14.6)	8 (17.0)	0.118
No	362 (78.2)	253 (75.7)	70 (85.4)	39 (17.0)	

Abbreviation: 25(OH)D: 25-hydroxyvitamin D, IQR: interquartile range, BC: blood count, MCV: Mean corpuscular volume, MCH: mean corpuscular hemoglobin, MCHC: mean corpuscular hemoglobin concentration, eGFR: estimated glomerular filtration rate, CRP: C-reactive protein. ^1^ Kruskal–Wallis test was used to compare continuous variables, and χ^2^ test or Fisher’s exact test was used to compare qualitative variables. Significant level *p*< 0.05.

**Table 3:** Anemia status, severity of anemia, and iron status according to 25(OH)D3 cut-off points.

	All Participants (n = 463)	25 (OH)D (ng/mL) Cut-Off Points			*p*-Value ^1^
		<20 (n = 334)	20–30 (n = 82)	>30 (n = 47)	
		N (%)			
**Anemia status (HB < 12 mg/dL)**					<0.001
Non-anemic	355 (76.7)	233 (65.6)	46 (13.0)	76 (21.4)	
Anemic	108 (23.3)	101 (93.5)	6 (5.6)	1 (11.0)	
**Anemia severity (n = 108)**					
Mild to moderate (10.9 < Hb < 12 g/dL)	101 (93.51)	94 (93.1)	6 (5.6)	1 (0.6)	0.771
Severe (HB < 8 g/dL)	7 (6.48)	7 (100)	0 (0.0)	0 (0.0)	
**Iron deficiency**					
No	331 (71.5)	225 (68.0)	43 (19.0)	63 (13.0)	
Yes	132 (27.6)	109 (82.6)	19 (14.4)	4 (3.0)	0.001
**Iron deficiency anemia**					
No	409 (88.3)	285 (69.7)	77 (18.8)	47 (11.5)	
Yes	54 (11.7)	49 (90.7)	5 (9.3)	0 (0.0)	0.003

Abbreviation: HB: hemoglobin. ^1^ χ^2^ test or Fisher’s exact test, significant level *p* < 0.05.

**Table 4:** Regression analysis for anemia, iron deficiency, and iron deficiency anemia according to vitamin D status.

	Unadjusted Model			Adjusted Model		
	*β* (*SE*)	OR (95% CI)	*p*-Value	*Β* (*SE*)	OR (95% CI)	*p*-Value
**Outcome: Anemia**						
**Vitamin D status**						
Sufficient–insufficient (>20 ng/mL)		Reference			Reference	
Deficient (<20 ng/mL)	2.17 (0.43)	8.79 (4.06, 23.00)	<0.001	1.97 (0.45)	7.17 (3.19, 19.28)	<0.001
**Physical activity intensity**						
Moderate to high		Reference			Reference	
Low	1.67 (0.31)	5.35 (2.88, 9.91)	<0.001	1.76 (0.34)	5.80 (2.96, 11.62)	<0.001
**Iron deficiency**						
No		Reference			Reference	
Yes	1.54 (0.23)	4.70 (2.95, 7.49)	<0.001	1.61 (0.26)	5.02 (2.99, 8.45)	<0.001
**Geographic localization**						
Rural		Reference			Reference	
Urban	0.50 (0.22)	1.64 (1.06, 2.55)	0.026	0.57 (0.25)	1.77 (1.07, 2.94)	0.021
**Sunscore categories (%)**						
Sufficient to high SES		Reference			Reference	
Insufficient to moderate SES	0.46 (0.22)	1.59 (1.03, 2.46)	0.034	0.31 (0.25)	1.33 (0.82, 2.17)	0.221
**Iron intake (mg/day)**	−0.07 (0.02)	0.93 (0.88–0.98)	0.008	−0.05 (0.02)	0.94 (0.88, 0.99)	0.041
**Outcome: Iron deficiency**						
**Vitamin D status**						
Sufficient–insufficient (>20 ng/mL)		Reference			Reference	
Deficient (<20 ng/mL)	0.75 (0.25)	2.12 (1.28, 3.50)	0.003	0.75 (0.26)	2.20 (1.32, 3.77)	0.007
**Age (years)**	−0.04 (0.01)	0.95 (0.92, 0.98)	0.009	−0.04 (0.01)	0.95 (0.92, 0.98)	0.012
**Non-heminic iron** **intake (mg/day)**	−0.04 (0.02)	0.95 (0.90, 1.00)	0.084	−0.05 (0.02)	0.95 (−1.10, 1.00)	0.037
**Education level**						
Illiterate		Reference			Reference	
Literate	−0.38 (0.18)	0.67 (0.46, 0.97)	0.037	−0.30(0.14)	0.74 (0.54, 0.99)	0.069
**Outcome: Iron deficiency anemia**						
**Vitamin D status**						
Sufficient–insufficient (>20 ng/mL)		Reference			Reference	
Deficient (<20 ng/mL)	1.42 (0.48)	4.16 (1.62, 10.71)	0.003	1.41 (0.48)	4.10 (1.73, 12.08)	0.004
**Iron intake (mg/day)**	−0.08 (0.03)	0.92 (0.86, 0.98)	0.013	−0.08 (0.03)	0.91 (0.85, 0.99)	0.030
**Geographic localization**						
Rural		Reference			Reference	
Urban	0.60 (0.29)	1.82 (1.02, 3.23)	0.039	0.55 (0.30)	1.74 (0.96, 3.15)	0.084
**Education level**						
Illiterate		Reference			Reference	
Literate	−0.397 (0.20)	0.67 (0.44, 1.00)	0.052	−0.38 (0.21)	0.68 (0.44, 1.03)	0.070

Abbreviations: β: Beta coefficient; SE: Standard Error; OR: Odds Ratio; SES: Sun Exposure Score; significance level: *p* < 0.05.

**Table 5:** Summary of ROC Analysis for VDD and covariates predicting anemia and iron status.

Outcome	Predictors	AUC	95% CI for AUC	*p*-Value for AUC	Optimal Threshold	Youden Index	Optimal Sensitivity	Optimal Specificity
Anemia	VDD	0.643	0.610–0.676	<0.001	0.175	0.285	0.944	0.341
	VDD, localization, ID, iron intake and physical activity, sun exposure	0.813	0.768–0.855	<0.001	0.269	0.528	0.722	0.806
ID	VDD	0.575	0.535–0.616	<0.001	0.250	0.151	0.833	0.317
	VDD, age, non-heminic iron intake, education level	0.654	0.604–0.704	<0.001	0.233	0.243	0.634	0.648
IDA	VDD	0.603	0.557–0.644	<0.001	0.093	0.206	0.907	0.298
	VDD, iron intake, localization, education level	0.712	0.643–0.776	<0.001	0.123	0.389	0.741	0.648

Abbreviations: VDD: Vitamin D deficiency, ID: iron deficiency, IDA: Iron deficiency anemia, AUC: Area under the curve, CI: confidence interval. Statistical significance, *p* < 0.05.

## Data Availability

The datasets generated and analyzed for the current study are available from the corresponding author upon reasonable request.

## Supplementary Materials

The following supporting information can be downloaded at: https://www.mdpi.com/article/10.3390/epidemiologia5040055/s1, Table S1: Predictors of vitamin D deficiency in study participants: logistic regression results.; Table S2: Linear regression analysis of vitamin D intake quartiles in association with 25(OH)D levels.
