# Supplementary material for: The Interplay Between Vitamin D Deficiency, Iron Status, and Anemia Risk in Moroccan Women of Reproductive Age: A Cross-Sectional Analysis
Source: Epidemiologia (Basel). 2024 Dec 19;5(4):805–27. doi: 10.3390/epidemiologia5040055 (PMC11675096; doi:10.3390/epidemiologia5040055)
Supplement: Supplementary file 1 [file epidemiologia-05-00055-s001.zip › epidemiologia-3214606-supplementary.pdf]

Table S1. Predictors of vitamin D deficiency in study participants : logistic regression results.

**Variables**	***β***	***SE***	**OR (95% IC)**	***p*-value**
ID	0.75	0.27	2.13 (1.25-3.62)	0.005
Sun exposure category (insufficient to moderate)	0.87	0.23	2.40 (1.53-3.77)	0.0001
obesity (BMI>30kg/m²)	0.21	0.09	1.24 (1.03-1.48)	0.0174
Age	-0.03	0.017	0.96 (0.93-0.99)	0.0361
Saison (spring-summer)	-0.52	0.22	0.58 (0.38-0.91)	0.0171

Table S2 : Linear regression analysis of vitamin D intake quartiles in association with 25(OH)D levels.

**Dependant variable 25(OH)D3**	**Quartiles of vitamin D intake** [d]	**B(*SE*)**	**t-Value**	**95%CI (LL)**	**95%CI (UL)**	**p-Value**
[a] Model 1	Q1	Reference				
	Q2	0.13(0.06)	1.99	0.00	0.26	0.046
	Q3	0.13(0.06)	1.96	0.00	0.26	0.050
	Q4	0.21(0.06)	3.24	0.08	0.34	0.001
[b] Model 2	Q1	Reference				
	Q2	0.11(0.06)	1.73	- 0.01	0.23	0.083
	Q3	0.10(0.06)	1.69	-0.01	0.23	0.091
	Q4	0.21(0.06)	3.33	0.08	0.33	0.001
[c] Model 3	Q1	Reference				
	Q2	0.08(0.06)	1.44	-0.03	0.20	0.150
	Q3	0.09(0.06)	1.56	-0.02	0.21	0.117
	Q4	0.16(0.06)	2.68	0.04	0.28	0.008

Abbreviation; Q: quartile of vitamin D intake; LL: lower limit, UL: upper limit

[a] Model 1: crude association between serum 25(OH)D concentrations and quartiles of vitamin D intake.

[b] Model 2: Model 1 + adjustment for physical activity, BMI, sun exposure score, eGFR, season, and sociodemographic characteristics.

[c] Model: Model 2 + adjustment for hemoglobin and ferritin levels.

[d] Quartiles of vitamin D intake ; Q1 : 0.2-1.67 µg/day ; Q2 : 1.68-2.74µg/day, Q3 : 2.75-4.40 µg/day ; Q4 : 4.41 -16 µg/day.
